# Failure of Achieving Tacrolimus Target Blood Concentration Might Be Avoided by a Wide Genotyping of Transplanted Patients: Evidence from a Retrospective Study

**DOI:** 10.3390/jpm10020047

**Published:** 2020-06-01

**Authors:** Giovanni Pallio, Natasha Irrera, Alessandra Bitto, Federica Mannino, Letteria Minutoli, Michelangelo Rottura, Socrate Pallio, Domenica Altavilla, Angela Alibrandi, Maria Concetta Marciano, Maria Righi, Carmen Mannucci, Vincenzo Arcoraci, Francesco Squadrito

**Affiliations:** 1 Department of Clinical and Experimental Medicine, University of Messina, Via C. Valeria, 98125 Messina, Italy; gpallio@unime.it (G.P.); nirrera@unime.it (N.I.); abitto@unime.it (A.B.); fmannino@unime.it (F.M.); lminutoli@unime.it (L.M.); mrottura@unime.it (M.R.); spallio@unime.it (S.P.); fsquadrito@unime.it (F.S.); 2 Department of Biomedical, Dental, Morphological and Functional Imaging Sciences, University of Messina, Via C. Valeria, 98125 Messina, Italy; daltavilla@unime.it (D.A.); mrighi@unime.it (M.R.); cmannucci@unime.it (C.M.); 3 Department of Economics Section of Statistical and Mathematical Sciences, University of Messina, Via dei Verdi, 98122 Messina, Italy; aalibrandi@unime.it; 4 Grande Ospedale Metropolitano: “Bianchi-Melacrino-Morelli”, Via Giuseppe Melacrino, 89124 Reggio Calabria, Italy; k.marciano25@tiscali.it

**Keywords:** pharmacogenetics, polymorphism, tacrolimus, immunosuppressant, therapeutic drug monitoring

## Abstract

Precise tacrolimus treatment in transplanted patients is achieved in the clinical setting by performing therapeutic drug monitoring (TDM) and consequently adjusting therapy. The aim of this study was to retrospectively analyze the variability in tacrolimus blood levels throughout 2 years of observation in 75 transplanted patients and to investigate if tacrolimus blood levels correlate with presence of genetic polymorphisms, thus modifying tacrolimus pharmacokinetics. CYP3A5*1 (G6986A), CYP3A4*1B (A392G), CYP3A4*22, ABCB1 (C3435T; C1236T; G2677A/T), SLCO1B1 (T521C), polymorphisms were analyzed. Based on the effect of their genotypes, patients were stratified into 5 groups: (1) reduced tacrolimus metabolism (RM), (2) increased metabolism (IM), (3) transporters polymorphisms (TM), (4) metabolism and transporter polymorphisms (AM) and (5) no mutations (Wild Type, WT). The percentage of the samples out of therapeutic range was significantly higher in the IM group than in the WT group (*p* = 0.001), as well as compared to the TM group (*p* = 0.004). Only IM pattern (*p* = 0.015) resulted as an independent predictor of number of tacrolimus blood levels out of therapeutic range. RM pattern (*p* = 0.006) was inversely related to the administered dose. Therefore, genotyping could become a standard practice before tacrolimus prescription thus decreasing side effects, increasing efficacy and reducing the economic burden for the national health system.

## 1. Introduction

Tacrolimus is a calcineurin inhibitor widely used as an antirejection drug for patients who underwent liver or kidney transplantation. Tacrolimus is a macrolide that binds the FK binding protein 12 (FKBP-12), thus forming a complex that in turn engages calcineurin. This binding prevents the dephosphorylation and nuclear translocation of nuclear factor of activated T-cells, inhibiting IL-2 production and T-lymphocyte activation [1]. Tacrolimus use in clinical practice is complicated by its narrow therapeutic index as well as its high pharmacokinetics variability in patients. For this reason, underexposure with risk of rejection, or overexposure with risk of toxicity may be detected. In particular, nephrotoxicity, hyperglycemia, neurotoxicity, and hypertension were observed following tacrolimus use in transplanted patients [2]. Therefore, current management of tacrolimus needs therapeutic drug monitoring (TDM) [3]. Blood therapeutic levels of tacrolimus should range between 4 and 10 ng/mL in kidney transplant patients [4] and drug concentrations are generally monitored 12 h following the administration of the drug, even if it has been demonstrated that drug levels measured in the following 6 h better correlate with the 12 h area under the concentration-time curve (AUC) in stable transplantation patients [5]. Unfortunately, TDM does not provide information on the starting dose used although it may be useful for adjusting the therapeutic regimen: indeed, individual differences in first pass metabolism have to be considered because they may delay reaching target blood concentrations with the initial selected dose. Tacrolimus pharmacokinetics is characterized by poor bioavailability (around 20%) [6]. The peak of concentration is usually reached within 2 h following oral administration; then, the drug is distributed in tissues and in the cellular fraction of blood [6]. Specifically, erythrocytes highly express tacrolimus receptor and this is the reason why tacrolimus concentration is about 15 times higher in whole-blood than the concentration found in plasma. The metabolism of tacrolimus mainly occurs in liver, but also gut and kidney may be involved. The reactions of demethylation and hydroxylation are mediated by the two hepatic and intestinal isoforms of CYP3A, CYP3A4 and CYP3A5; the resulting metabolites may be found in blood at low concentrations, thus showing a lesser pharmacological activity than tacrolimus itself. After metabolism, the drug is mainly excreted in bile (around 90%). Tacrolimus is also a substrate for the multidrug efflux transporter P-glycoprotein which is encoded by the ABCB1 gene and is expressed on epithelial, endothelial cells and lymphocytes [7]. Variations in tacrolimus pharmacokinetics among patients are related to differences and alterations in (i) the genes encoding the enzymes that metabolize for CYP3A5 and CYP3A4, (ii) the drug transporter ABCB1 and SLCO1B1 [8,9]. Enzymes that belong to the cytochrome P450 (CYP3A) family are responsible for the oxidative metabolism of tacrolimus; among them, CYP3A4 and CYP3A5 play a relevant role. Indeed, expression of CYP3A5 has been found to be largely determined by genetic polymorphisms, the most important variant being CYP3A5*3, which causes the lack of the enzyme. The production of a truncated protein which is not functionally active is related to the variation of the sequences A→G at the level of the nucleotide 6986 placed in intron 3 of the CYP3A5 gene. As a consequence, the blood levels of drugs metabolized by this CYP could significantly increase with an increased risk of adverse reactions. On other hand, the G6986A (CYP3A5*1) allele leads to an increased expression of the protein with improved metabolism of drugs and reduced drugs blood level that could lead to therapeutic failure. In accordance with functional role of these polymorphisms, patients that show the variant allele CYP3A5*3 are the so-called “CYP3A5 non-expressers”, whereas those that show the variant allele CYP3A5*1 allele encode for functional CYP3A5 enzymes and are considered as “CYP3A5 expressers” [10]. The frequencies of CYP3A5 polymorphisms may depend on different ethnicity. In fact, CYP3A5*1 allele is differently distributed in the individuals belonging to different ethnic groups, with a percentage between 5% and 15% in the Caucasian population [11]. Other important polymorphisms studied in the metabolism of tacrolimus occur on gene that encode for CYP3A4. An A>G replacement at position 392 causes the production of a variant allele, CYP3A4*1B, that shows an increased enzymatic activity with improved drugs metabolism. The reported frequency for Caucasian population is between 2% and 10% [12,13,14]. Furthermore, CYP3A4*22 SNP, which is expressed in Caucasians (5%), is responsible for a reduced expression of CYP3A4 related to increased drugs blood levels. CYP3A4*22 SNP in association with the lack of CYP3A5 expression may increase tacrolimus concentrations over the therapeutic levels, thus raising its toxicity [15]. Moreover, P-glycoprotein (P-gp), encoded by the ABCB1 gene, showed several SNPs mainly 1199G>A, 2677G>T/A, 3435C>T and 1236C>T in humans. The presence of these SNPs, both the presence of only one or more than one together, significantly reduces P-gp function and increases tacrolimus bioavailability [16,17]. ABCB1 expression is influenced by ethnicity and the combined haplotype (2677G>T/A, 3435C>T, 1236C>T) is present in approximately 35% of Mexican Americans, 32% of Caucasians, 27% of Asian Americans and 5% of African Americans [18,19]. Moreover, oral bioavailability of tacrolimus may be influenced by the organic anion transporting polypeptide-C (encoded by SLCO1B1 gene), which is its transporter specifically expressed in liver and contributes to the biliary excretion of tacrolimus. 521T>C SNP in the SLCO1B1 gene significantly increases tacrolimus concentrations in blood, whereas 388A>G SNP significantly reduces tacrolimus concentrations [17]. Due to these significant differences both in the expression and consequently in the function of CYP3A4, CYP3A5, ABCB1 and SLCO1B1 genes caused by SNPs, a wide pharmacogenetics approach before transplantation may be helpful to better predict tacrolimus blood concentrations, to reduce toxicity after transplantation and to further optimize the individualization of tacrolimus dosing in transplanted patients. However, very few studies have simultaneously analyzed all the complex pattern of the SNPs causing inter-individual tacrolimus variability. Therefore, the purpose of this study was to analyze some SNPs and to investigate whether they might cause frequent out of range values and high variability in tacrolimus blood concentration.

## 2. Results

### 2.1. Patients’ Characteristics

Basic characteristics of the 75 patients included in this study were summarized in Table 1 and Table S1. All patients with mutations that increase or reduce metabolism also had at least one mutation on transporters. Only one patient had mutations on all genes (AM) and was therefore excluded from the analyses. Median tacrolimus blood levels and tacrolimus administered dose were significantly higher in adults than in children (*p* < 0.001 and *p* < 0.001, respectively). No differences by gender or allelic mutations were shown. Moreover, our study did not include patients in the early stage (1–6 months) after organ transplant and therefore no patient had renal insufficiency, warm ischemia time, delayed graft function, and chronic allograft dysfunction. All patients enrolled in our study had a kidney stable transplant and none experienced severe changes in renal function. Therefore, dosage adjustments were almost always secondary to the need to comply with the recommendations relating to maintaining blood levels within the therapeutic range and were guided by the information obtained from the therapeutic drug monitoring.

### 2.2. Tacrolimus Blood Levels

**Figure 1:**
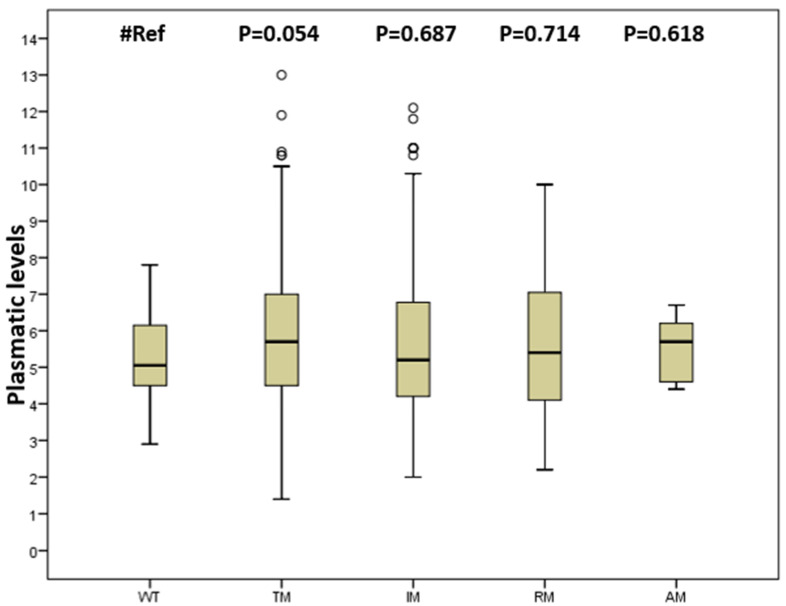
Tacrolimus blood levels stratified by group of gene mutation. WT: wild type; IM: mutations that increase metabolism; RM: mutations that decrease metabolism; TM: transporters mutations; AM: at least one mutation in all gene groups (IM, RM, TM).

Median tacrolimus blood levels were 5.1 (4.6–6.1) in wild type (WT) subjects. No difference was observed between WT group and other groups in tacrolimus blood levels (Figure 1). At least one blood value out of range was observed, mostly in patients with allelic mutations (Table 2). A total of 99 samples out of therapeutic range, 16 higher and 83 lowers, were found. The percentage of samples out range was significantly higher in the increased metabolism (IM) group (25.9%) than in the WT group (5.4%; *p* = 0.001), as well as compared to the transporters polymorphisms (TM) group (15.0%; *p* = 0.004) (Table 2).

Age of patients as well as gender did not influence the probability of being out of therapeutic range. IM pattern (B = 0.213 (0.041/0.384); *p* = 0.015) resulted as an independent predictor of number of tacrolimus blood levels out of therapeutic range, whereas reduced tacrolimus metabolism (RM) and TM patterns were not related to the increased risk of being out of therapeutic range. The presence of IM mutation (B = 0.206 (0.031/0.381); *p* = 0.021) influenced the probability of being out of therapeutic range also independently from the administered dose (Table 3).

### 2.3. Tacrolimus Dose

Tacrolimus median dose was 3.5 (2.5–4.0) in WT group and 2.0 (1.5–5.0), 3.0 (2.0–4.5), 0.5 (0.5–0.5), in TM, IM, RM, respectively. Administered median dose was significantly lower in RM (*p* < 0.001) and TM (*p* = 0.043) groups than in WT group. No significant difference was shown between WT and IM groups (*p* = 0.574).

Patient gender did not influence the drug dose, whereas age (b = 0.287 (0.141/0.433); *p* < 0.001) modified the dosage regimen. Only the presence of RM mutation (b = −0.513 (−0.876/−0.150); *p* = 0.006) was inversely related to the administered dose. No significant correlation was observed between the other groups of mutations and the tacrolimus dose (Table 4). 

The post-hoc power calculation, including 75 patients as sample size stratified in four different groups and with an α error probability = 0.05, showed an effects size f = 0.3978 and a power (1-β error probability) = 81.3%.

## 3. Discussion

Inappropriate management of transplanted patients causes an incorrect therapeutic approach and an economic burden to the health care system. The results of the present study suggest that failure of achieving tacrolimus target blood concentration might be avoided by a wide genotyping of transplanted patients. Genotyping patients for the genes involved in tacrolimus metabolism and transport might allow the identification of subgroups of patients with drug concentration out of the therapeutic range.

Indeed, the daily practice of drug monitoring reveals wide between-individual variability in tacrolimus pharmacokinetics and particularly in the dose required to achieve target blood concentrations. Polymorphisms of the genes coding for biotransformation enzymes (CYP3A4 and CYP3A5) and drug transporters (ABCB1 and SLCO1B1) were investigated to date. CYP3A5 expression may be dependent on genetic polymorphisms and CYP3A5 polymorphisms differently occur according to ethnicity [10]. CYP3A5*1 allele is distributed in 5–15% of Caucasians, in the present study, 12.0% of patients showed mutation according to the reported frequency.

Patients with one or more CYP3A5*1 allele showed a reduced tacrolimus concentration to dose ratio compared to non-expressors CYP3A5*3 in kidney, liver, lung, and heart transplant recipients [11]. A study carried out on 209 patients subjected to kidney transplant demonstrated that individuals with one or more CYP3A5*1 allele showed higher tacrolimus dose requirements and lower drug levels than CYP3A5*3 [20]. Another study carried out on 35 patients with kidney transplant showed that tacrolimus concentration/dose ratios were significantly reduced in patients expressing CYP3A5*1 allele compared to patients with CYP3A5*3 genotype, for six weeks following transplant [21]. Moreover, twofold lower dose-normalized tacrolimus blood concentrations were found in renal transplanted patients with CYP3A5*1 compared to patients with a CYP3A5*3 profile [22]. In fact, tacrolimus dose requirements were significantly increased in transplant patients with the CYP3A5*1 genotype [23]. Moreover, A>G substitution in the gene that encodes for CYP3A4 is a polymorphism involved in tacrolimus metabolism; this alteration causes a variant allele characterized by an increased enzymatic activity, known as CYP3A4*1B. The frequency of the A>G substitution has been observed in Caucasians with a percentage of 2–10% [14]. In our setting the A>G substitution was observed in 22.7% of patients; the obtained result is higher than observed in Caucasian population. However, the role of this SNP in the metabolism of different drugs has not been fully understood [24,25,26]. Moreover, a correlation between the CYP3A4*1B allele and tacrolimus pharmacokinetics has not been observed [27], nor has the influences of the CYP3A5 6986A>G SNP and ABCB1 polymorphisms [28].

In our study, the number of the samples out of range was significantly higher in IM group than in WT or in TM group, due to low blood values of tacrolimus. Moreover, the presence of IM genetic mutation is directly related to the number of out of range samples. However, no correlation was observed between IM genetic polymorphisms and tacrolimus dose administered. Failure to increase the dose of tacrolimus administered could justify the observed results.

The CYP3A4*22 C>T polymorphism was recently identified and studied in transplant patients: renal transplant patients, Caucasians, with T alleles needed lower tacrolimus doses compared to individuals with wild-type C allele [29]. This SNP is relatively frequent in Caucasians (2.5–6.9%) and in our sample the presence of this polymorphism was 6.7% according to the reported frequency in Caucasians. RM group did not show a significantly different number of samples out of range compared to WT. Moreover, the presence of RM genetic mutation was not related to the number of tacrolimus samples out of range, as assessed using the generalized linear model (GLM). On the contrary, an inverse relationship between RM genetic polymorphisms and tacrolimus dose administered was observed. The reduced dose of tacrolimus administered could justify the obtained observed results.

As for as CYP3A4 and CYP3A5, several SNPs have been identified in ABCB1 gene. The SNP in exon 26 (C3435T) is in strict imbalance with two other SNPs located in exon 12 (C1236T) and exon 21 (G2677T/A), respectively [30]. These three variant alleles often exist together, thus demonstrating that they might be connected at a genetic level. This haplotype occurs in 32% of Caucasians [18]. According to the previous papers, this haplotype was present in 28% of our sample. The association between tacrolimus pharmacokinetic parameters and the ABCB1 genotype is controversial. Indeed, some studies did not demonstrate an association [3], whereas others described a significant connection between ABCB1 polymorphisms and tacrolimus dose requirements in kidney transplant patients [31,32]. Moreover, it could be important to consider that the association of SNPs might be more predictive than testing for single polymorphic sites [27]. Different studies showed that a correlation between ABCB1 polymorphisms and tacrolimus dosing requirements did not exist in patients subjected to kidney transplant [33,34]. López-Montenegro Soria et al. found that patients with renal transplant and with a wild type ABCB1-3435CC genotype showed up to 40% lower concentration/dose ratios than individuals with variant alleles [21]. The organic anion transporting polypeptide 1B1 (OATP1B1), encoded by SLCO1B1 gene, is a tacrolimus transporter which is expressed in liver and is involved in the biliary excretion of tacrolimus. Both 388A>G and 521T>C polymorphisms in SLCO1B1 gene influenced tacrolimus levels in blood. In particular, patients with the 521 polymorphism showed an increased mean of tacrolimus concentrations in blood [17]. We have grouped patients with a SNP on transporter (ABCB1 or SLCO1B1) in TM group (patients with mutation on transporter) and, according to previous studies, we observed no difference in tacrolimus blood concentration compared to WT group. Moreover, the presence of mutations did not influence the probability of being out of therapeutic range as well as no correlation has been found between a transporter mutation and the tacrolimus administered dose. All patients with a SNP on CYP3A4 had also a SNP on transporter (ABCB1 or SLCO1B1); the same was observed for patients with a SNP on CYP3A5. However, the concomitant presence of a SNP on transporter was not enough to increase frequency of out of range tacrolimus blood levels in the RM group, as previously reported [29]. Moreover, age and gender did not result in predictive factors of being out of therapeutic range and only age significantly correlated to the tacrolimus dose. Although the present study is worthy of interest, a low number of subjects was enrolled and several genetic mutations coexisted, causing opposite effects in many patients. Nevertheless, the obtained results demonstrate that genotyping transplanted patients might be useful to improve the management of patients treated with tacrolimus. Indeed, TDM seems to be not enough to induce the dose adjustment, in particular whether the dose has to be increased. Sub-range tacrolimus blood levels were mostly observed in patients with genetic mutations that increased metabolism, but the administered dose of tacrolimus was not sufficiently increased, and was similar to that of wild type patients. On the contrary, the administered dose of tacrolimus was inversely related to the presence of mutations reducing metabolism, and no relationship between RM genotype and number of out of range blood levels was shown. These findings suggest that, without information on the metabolic pattern of patients, physicians reduce or maintain drug doses instead of increasing them, in order to prevent drug toxicity. However, some limitations of our study should be taken into account. Indeed, even if the simultaneous evaluation in the same patient of all the relevant polymorphisms involved in tacrolimus pharmacokinetic can represent the strength of the study, at the same time it could require, given the not high frequency of these mutations in the general population, the enrollment of a greater number of patients. Furthermore, notwithstanding dose adjustment, several patients continued to show tacrolimus blood values outside of the therapeutic range. This data might be due to the presence of genetic mutations that influence metabolism and distribution of tacrolimus but also other different conditions, analyzed on a case-by-case basis, should be considered. In conclusion, the present study suggests that genotyping transplanted patients should become a standard practice before tacrolimus prescription in order to reduce side effects, increase efficacy and reduce the costs for the national health system.

## 4. Materials and Methods

### 4.1. Patients and Data Collection

A total of 173 patients referring to the Toxicology and Drug Monitoring Unit of Messina University Hospital were enrolled from January 2017 to December 2018. In total, 75 subjects were included in the study. Exclusion criteria were as follows: patients with other type of transplant, in the early stage (6 months) of treatment after transplant, in therapy with other immunosuppressant (corticosteroid or mycophenolate mofetil), with previous transplant rejection, in therapy with drugs that increase tacrolimus blood levels (macrolides, antimycotics, calcium channel blockers), in therapy with drugs that reduce tacrolimus blood levels (antibiotics, antiepileptic), with a concomitant transplant, with impaired vital functions. Inclusion criteria were as follows: patients with Kidney stable transplant (almost 6 months), receiving tacrolimus as a primary immunosuppressant. All patients included in the study were genotyped as described below and, according to the effect of their genotypes, they were stratified into 5 groups: patients with (1) reduced tacrolimus metabolism (RM), (2) increased metabolism (IM), (3) transporters polymorphisms (TM), (4) metabolism and transporter polymorphisms (AM) and (5) no mutations (Wild Type, WT). Informed consent was obtained from all the subjects or their legal representative. All subjects gave their informed consent for inclusion before they participated in the study. Tacrolimus dose was collected the same day of blood sampling and each patient was monitored at least once in two months for two years. The study was carried out in accordance with the Declaration of Helsinki, and the protocol was approved by the Ethics Committee of Messina, project identification code N° 47/17 17/07/2017.

### 4.2. Tacrolimus Dosage, DNA Extraction and Real-Time PCR

Venous blood was collected in 3 mL tubes containing EDTA as anticoagulant. Tacrolimus concentrations were assayed in whole blood using the Siemens Dimension EXL-200 system. Genomic DNA was isolated from whole blood, cells were lysed in DNA buffer containing 10 mM Tris-HCl pH 7.4, 100 mM NaCl, 1 mM EDTA, 1 mM MgCl2. After centrifugation nuclei were lysed by the combined action of 10% SDS and proteinase K at 37 °C overnight. The cell debris from lysis, mainly protein, were captured by the organic solvents: phenol and chloroform. DNA was washed with 70% ethanol and resuspended in an adequate volume of RNAse/DNAse free water. DNA concentration in each sample was spectrophotometrically determined and stored for genotyping assay. For analysis of genotypes a common primer and two allele-specific primers were designed, with the 3’ base of each specific primer corresponding to either allele wild type or mutant, respectively (Table 5). Depending on the sample genotype, either one or the other or both allele-specific primer(s) can amplify the target sequence. To enhance the specificity of the assay, a mismatch at the third 3’ base of both specific primers was employed [35]. Two PCR amplification reactions were set up for each sample. The component of the two reactions was identical except for the respective allele-specific primers, one matching to wild type and the other to mutant. The real-time PCR was carried out on a QuantStudio6 Flex (Applied Biosystems, Foster City, CA, USA) by a 20 µL reactions mixture of 10 µL BrightGreen qPCR MasterMix (abm, Richmond, BC, Canada), 10 µM of each primer (0.6 µL), 15 ng of genomic DNA (1 µL) and DEPC water to obtain a final volume of 20 uL. The amplification conditions were as follows: 1 cycle of enzyme activation at 95 °C for 10 min, followed by 40 cycles of denaturation at 95 °C for 15 s and annealing/extending at 60 °C for 1 min. The binding efficiency of allele-specific primer to target sequence was high when they matched well at the 3’ end of the primer, resulting in efficient amplification with smaller Ct. In contrast, the efficiency of amplification was low with mismatch between the primer and the target sequence, resulting in larger Ct. Allele discrimination of genes was successfully achieved based on the difference between the Ct values from the two reactions of each sample.

### 4.3. Statistical Analysis

Descriptive statistical analyses were performed to evaluate basal demographic and clinical characteristics of all patients. All results were expressed as median with interquartile range (Q1–Q3) for continuous variables, and absolute frequencies and percentages for categorical variables. Since most of the variables were not normally distributed (as resulted by Kolmogorov–Smirnov test), the nonparametric approach was used. Accordingly, the Mann–Whitney (comparison between two independent groups of patients) U test for continuous variables and the Pearson Chi Square test for categorical variables were applied. For each patient, all blood drug values, monitored during the study period, were recorded and the number of samples out of range was analyzed. Moreover, patients were stratified according to the occurrence of at least one tacrolimus blood level out of range or not. The out of range events were calculated as the ratio between the number of out range blood samples and the total number of blood samples, per patient. A generalized linear model (GLM) was estimated to identify significant predictors of out of range events. The considered predictors were as follows: sex and age (adult or child), transporters mutation (yes or no), reducing metabolism mutation (yes or no), increasing metabolism mutation (yes or no). The model was also estimated adjusting for administered dose. Moreover, a GLM was estimated to identify significant predictors of administered dose and the same covariates were considered. The results were expressed for each covariate as B coefficient with 95% Confidence Interval (95% C.I.). A post-hoc power calculation (F tests in fixed effects one-way ANOVA) was performed on tacrolimus blood levels, considering the whole sample size and assuming four different groups with an α error probability = 0.05. A *p*-value smaller than 0.05 was considered statistically significant. Statistical analyses were performed using SPSS Statistics for Windows v22.0 (SPSS, Inc, Chicago, IL, USA) and G Power 3.1 (Faul, F., Erdfelder, E., Buchner, A., and Lang, 2009).

## Figures and Tables

**Table 1:** Patient’s characteristics.

	Patients		Adults		Children		*p*-Value
	n (75)	(%)	n (55)	(%)	n (20)	(%)	
**TDM Median [Q1–Q3]**	5.4 (4.4–6.8)		5.7 (4.6–7.0)		5.0 (4.0–6.1)		<0.001
**Dose Median [Q1–Q3]**	3.0 (1.5–4.5)		3.0 (2.0–5.5)		2.0 (1.0–4.0)		<0.001
**Female**	25	33.3	17	30.9	8	40.0	0.663
**Male**	50	66.7	38	69.1	12	60.0	--
**CYP3A5 (G6986A) Mut**	9	12.0	7	12.7	2	10.0	0.748
**CYP3A4 (A392G) Mut**	17	22.7	12	21.8	5	25.0	0.771
**ABCB1 (C3435T) Mut**	55	73.3	40	72.7	15	75.5	0.844
**ABCB1 (C1236T) Mut**	22	29.3	15	27.3	7	35.0	0.516
**ABCB1 (G2677T/A) Mut**	47	62.7	32	58.2	15	75.0	0.183
CYP3A4*22 Mut	5	6.7	4	7.3	1	5.0	0.727
**SLCO1B1 (T521C) Mut**	19	25.3	13	23.6	6	30.0	0.575
**WT**	5	6.7	4	7.3	1	5.0	--
**TM**	45	60.0	33	60.0	12	60.0	0.747
**IM**	20	26.7	14	25.5	6	30.0	0.656
**RM**	4	5.3	3	5.45	1	5.0	0.858
**AM**	1	1.3	1	1.8	-	-	

WT: wild type; IM: mutations that increase metabolism; RM: mutations that decrease metabolism; TM: transporters mutations; AM: at least one mutation in all gene groups (IM, RM, TM).

**Table 2:** Out of range tacrolimus blood levels stratified by group of gene mutation.

	N Patients	At Least Once Out of Range	(%)	N of Samples	N of Samples Out of Range	% of Samples Out of Range	*p*-Value vs. WT	*p*-Value vs. TM
**WT**	5	1	20.0	56	3	5.4	--	
**TM**	45	25	55.6	361	54	15.0	0.052	--
**IM**	20	14	70.0	143	37	25.9	0.001	0.004
**RM**	4	3	75.0	28	5	17.9	0.066	0.169
**AM**	1	0	0.0	5	0	0.0		

WT: wild type; IM: mutations that increase metabolism; RM: mutations that decrease metabolism; TM: transporters mutations; AM: at least one mutation in all gene groups (IM, RM, TM).

**Table 3:** Predictive factors of being out of therapeutic range.

	Not Normalized by Dose		Normalized by Dose	
Variable	B [95% CI]	*p*-Value	B [95% CI]	*p*-Value
**Sex (M)**	0.003 (−0.087/0.093)	0.949	0.005 (−0.087/0.097)	0.918
**Age (Child)**	−0.096 (−0.192/0.001)	0.051	−0.094 (−0.208/0.020)	0.104
**TM**	0.100 (−0.060/0.259)	0.221	0.113 (−0.047/0.274)	0.167
**IM**	0.213 (0.041/0.384)	0.015	0.206 (0.310/0.381)	0.021
**RM**	0.123 (−0.118/0.363)	0.317	0.099 (−0.167/0.365)	0.467
**Dose**	--	--	−0.011 (−0.034/0.012)	0.364

IM: mutations that increase metabolism; RM: mutations that decrease metabolism; TM: transporters mutations. B: Unstandardized Coefficient beta; CI: Confidence Interval.

**Table 4:** Relation between patient’s characteristics and tacrolimus dose.

Variable	B [95% CI]	*p*-Value
**Sex (M)**	−0.086 (−0.213/0.041)	0.183
**Age**	0.287 (0.141/0.433)	<0.001
**TM**	−0.015 (−0.236/0.205)	0.891
**IM**	0.096 (−0.143/0.335)	0.433
**RM**	−0.513 (−0.876/-0.150)	0.006

IM: mutations that increase metabolism; RM: mutations that decrease metabolism; TM: transporters mutations.

**Table 5:** Primers used for genotyping.

RS	Type of Primer	Sequence
CYP3A4*1B (A392G) RS2740574	WT special primer	5’ CAGCCATAGAGACAAGGGGAA 3’
	MUT special primer	5’ AGCCATAGAGACAAGGGGAG 3’
	Common primer	5’ GGAAGAGGCTTCTCCACCTTG 3’
CYP3A5*3 (A698G) RS776746	WT special primer	5’ GGTCCAAACAGGGAAGAGAAAT 3’
	MUT special primer	5’ TGGTCCAAACAGGGAAGAGAAAC 3’
	Common primer	5’ AGTCCTTGTGAGCACTTGATG 3’
**ABCB1 (C3435T) RS1045642**	WT special primer	5’ TGGTGTCACAGGAAGAGTTC 3’
	MUT special primer	5’ GTGGTGTCACAGGAAGAGTTT 3’
	Common primer	5’ AACCCAAACAGGAAGTGTGG 3’
**ABCB1 (C1236T) RS1128503**	WT special primer	5’ TCCTGGTAGATCTTGAAGCGC 3’
	MUT special primer	5’ TCCTGGTAGATCTTGAAGCGT 3’
	Common primer	5’ AGATGTGCAATGTGACTGCTG 3’
**ABCB1 (G2677T) RS2032582**	WT special primer	5’ AGTTTGACTCACCTTCCCTGC 3’
	MUT special primer	5’ AGTTTGACTCACCTTCCCTGA 3’
	MUT special primer	5’ GTTTGACTCACCTTCCCTGT 3’
	Common primer	5’ GGTTCCAGGCTTGCTGTAATT 3’
CYP3A4*22 (C15389T) RS35599367	WT special primer	5’ AGTGTCTCCATCACACCCTGC 3’
	MUT special primer	5’ CAGTGTCTCCATCACACCCTGT 3’
	Common primer	5’ CCCAAAGACGCTGAGTGGAGAA 3’
**SLCO1B1 (T521C) RS4149056**	WT special primer	5’ TCCACGAAGCATATTACCCATGATCG 3’
	MUT special primer	5’ CCACGAAGCATATTACCCATGATCA 3’
	Common primer	5’ CAGCCATGAGGAACTATGAGTCCA 3’

## Supplementary Materials

The following are available online at https://www.mdpi.com/2075-4426/10/2/47/s1, Table S1: Patient’s characteristics by different genotype
