# Supplementary material for: Failure of Achieving Tacrolimus Target Blood Concentration Might Be Avoided by a Wide Genotyping of Transplanted Patients: Evidence from a Retrospective Study
Source: J Pers Med. 2020 Jun 1;10(2):47. doi: 10.3390/jpm10020047 (PMC7354451; doi:10.3390/jpm10020047)
Supplement: Supplementary file 1 [file jpm-10-00047-s001.pdf]

Table S1: Patient's characteristics by different genotype.

	WT*	TM	*P*-value	IM	*P*-value	RM	*P*-value
	**n 5**	**n 45**		**n 20**		**n 4**	
**Female (%)**	2 (40.0)	16 (35.6)	0.844	6 (30.0)	0.668	0 (0.0)	0.151
**Male (%)**	3 (60.0)	29 (64.4)		14 (70.0)		4 (100.0)	
**Children (%)**	1 (20.0)	12 (26.7)	0.747	6 (30.0)	0.656	1 (25.0)	0.858
**Adults (%)**	4 (80.0)	33 (73.3)		14 (70.0)		3 (75.0)	
**Tacrolimus dose** median (Q1–Q3**)**	3.5 (2.5–4.0)	2.0 (1.5–5.0)	0.043	3.0 (2.0–4.5)	0.574	0.5 (0.5–0.5)	<0.001
**Tacrolimus blood levels** median (Q1–Q3**)**	5.1 (4.5–6.1)	5.7 (4.5–7.0)	0.054	5.3 (4.2–6.9)	0.687	5.5 (4.1–7.1)	0.714

WT: wild type; IM: mutations that increase metabolism; RM: mutations that decrease metabolism；

TM: transporters mutations； *WT group is the reference one in different comparisons
